# Lung_PAYNet: a pyramidal attention based deep learning network for lung nodule segmentation

**DOI:** 10.1038/s41598-022-24900-4

**Published:** 2022-11-25

**Authors:** P. Malin Bruntha, S. Immanuel Alex Pandian, K. Martin Sagayam, Shivargha Bandopadhyay, Marc Pomplun, Hien Dang

**Affiliations:** 1 Department of Electronics and Communication Engineering, Karunya Institute of Technology and Sciences, Coimbatore, India; 2 Department of Computer Vision & Deep Learning, Orbo.Ai, Mumbai, India; 3 Department of Computer Science, University of Massachusetts Boston, Boston, MA USA; 4 Faculty of Computer Science and Engineering, Thuyloi University, Hanoi, Vietnam

**Keywords:** Computational biology and bioinformatics, Mathematics and computing

## Abstract

Accurate and reliable lung nodule segmentation in computed tomography (CT) images is required for early diagnosis of lung cancer. Some of the difficulties in detecting lung nodules include the various types and shapes of lung nodules, lung nodules near other lung structures, and similar visual aspects. This study proposes a new model named Lung_PAYNet, a pyramidal attention-based architecture, for improved lung nodule segmentation in low-dose CT images. In this architecture, the encoder and decoder are designed using an inverted residual block and swish activation function. It also employs a feature pyramid attention network between the encoder and decoder to extract exact dense features for pixel classification. The proposed architecture was compared to the existing UNet architecture, and the proposed methodology yielded significant results. The proposed model was comprehensively trained and validated using the LIDC-IDRI dataset available in the public domain. The experimental results revealed that the Lung_PAYNet delivered remarkable segmentation with a Dice similarity coefficient of 95.7%, mIOU of 91.75%, sensitivity of 92.57%, and precision of 96.75%.

## Introduction

Cancer is one of the deadliest diseases worldwide, irrespective of the socioeconomic status of the people involved^1^. There are many types of cancer, including brain, pancreatic, lung, breast, stomach, and head and neck cancers. Among the various cancer types, lung cancer has the highest incidence and mortality rates worldwide^2^. The leading method for the early detection of lung cancer is to obtain computed tomography (CT) images of the entire chest region and analyze the same for any abnormality. Trained doctors can examine CT images taken with the help of X-rays and lung nodules, if any, can be detected. As the number of cases increases every year, this analysis has become a herculean task and has taken a toll on the healthcare system. Computer-aided diagnosis (CAD) systems have been established to help physicians manage lung cancers. The focus of existing CAD systems is to detect lung nodules in CT images. The lung nodule detection algorithm evaluates CT scans and predicts the location of suspicious nodules in the bounding boxes. However, simply having a bounding box was insufficient. To accurately predict the chance of malignancy, radiologists must measure the change in nodule size in clinical settings, which necessitates the manual delineation of nodule boundaries^3^. Radiologists need manually segmented nodules at the pixel level, which takes time because nodules vary in a variety of parameters such as size (3–30 mm in diameter), morphology, brightness, and compactness. Therefore, it is vital to develop a CAD system for accurate and reliable nodule segmentation.

Lung nodule segmentation involves extraction of a nodule region with a boundary from the lung parenchyma. In addition, it is a challenging task for radiologists to find nodule regions when they are attached to lung vessels, lung walls, and other internal structures.

**Figure 1:**
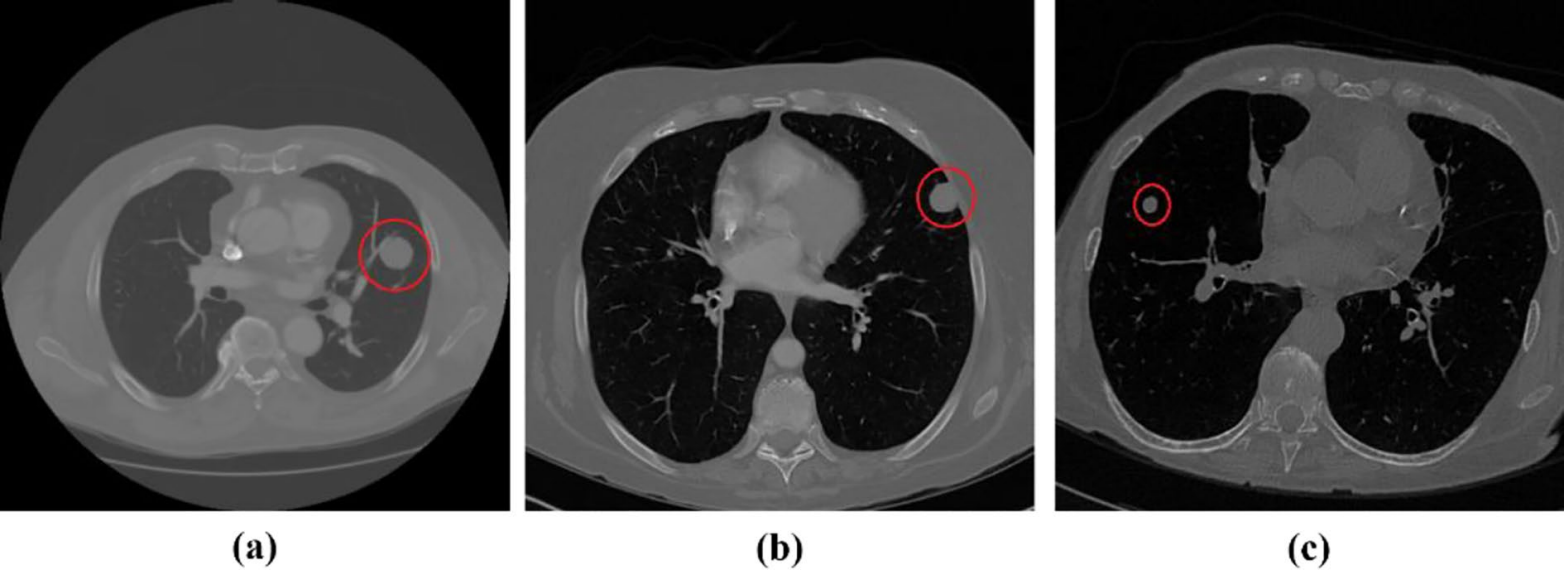
Different Nodule Types (**a**) Juxta-Vascular Nodule (**b**) Juxta-Pleural Nodule.(**c**) Well-Circumscribed nodule^4^.

Lung nodules can be classified based on their position in the lungs. Lung nodules that are not attached to any nearby structures are well-circumscribed. Juxta-pleural nodules are attached to the lung parenchyma and juxta-vascular nodules are affixed to the blood vessels. Figure 1 illustrates the various types of lung nodules obtained from the LIDC-IDRI dataset^4^. Depending on the malignancy factor, there may be benign (non-cancerous) or malignant (cancerous) lung nodules. It is necessary to segment the nodule carefully because it is critical to determine the malignancy factors.

Although many image-processing-based segmentation techniques^5–7^ have been applied to lung nodule segmentation, there is no generalized segmentation framework for segmenting various lung nodules using a single technique. Techniques suitable for segmenting well-circumscribed nodules may not be suitable for segmenting juxta-pleural or juxta-vascular nodules. This study focuses on end-to-end data-driven deep learning approaches to segment different types of lung nodules.

## Related work

Scientists have attempted to construct an exceedingly accurate, effective, and automatic lung nodule segmentation system that can help doctors to segment lung nodules. These attempts have been categorized into two major categories: image processing-based models and deep learning-based models.

Image processing models include morphology operations, region growing algorithms, and energy optimization techniques are the most frequently used methods. In morphology-based methods, researchers employed morphological opening operation and connected component selection methods to remove the vessels attached with nodules. However, separating lung nodules with extensive contact areas with other lung structures is challenging with the fixed size morphological template. As a result, more complicated morphological processes combining shape assumptions have been introduced.

Kuhnigk et al.^8^ found that blood vessel radii decreased as they evolved towards the perimeter of the lungs. Moreover, they recommended the use of rolling ball filters in combination with a rule-based analysis of juxta-pleural nodules. The selection of the morphological template size is a significant challenge for morphological approaches because it is difficult to identify an appropriate template for the morphology of diverse nodule sizes. The performance measure used in this work is median error and it was 3.1%.

Dehmeshki et al.^9^ developed a shape-based hypothesis to extract nodules from the lung wall. Here the segmentation method depended on sphericity oriented contrast region growing on the fuzzy connectivity map and the segmentation accuracy is 84%. Kubota et al.^10^ created a probability map to represent the probability of every pixel belonging to a nodule based on the local gray level. Diciotti et al.^11^ defined a semi-automatic technique based on region-growing for the 3D-segmentation of lung nodules in spiral CT images.

Zhou et al.^12^ introduced a fully automatic lung segmentation method for juxta-pleural nodules. A nonlinear anisotropic diffusion filtering method was employed to reduce image noise. The thoracic region was extracted using thresholding, 2D hole filling, and the largest connected-component search method. The lung parenchyma was separated using a fuzzy c-means algorithm, region-growing algorithm, and dynamic programming approach. An algorithm based on an adaptive curvature threshold has been proposed to include juxta-pleural nodules in the lung parenchyma. The average FP error was 1.89%, the average FN error was 2.39%, and the average volumetric overlap fraction was 95.81%.

Farag et al.^13^ adopted level set and shaped prior hypotheses to remove nodules from the lung wall. Boykov and Kolmogorov^14^ proposed a graph cut algorithm for lung nodule segmentation by outlining the problem as a maximum-flow optimization problem. Using non-parametric mean shift clustering, Ye et al.^15^ created a grey level and shape map using a variation of the graph cut approach.

For nodule segmentation, researchers have employed classification models associated with high features in machine learning methods. Wu et al.^16^ created a set of shape and texture features to represent voxels. Subsequently, a conditional random field (CRF) model for voxel classification was developed. Lu et al.^17^ created spatial image features such as translational and rotational invariant features, in which voxels from various nodules were mapped onto the same universal space.

Messay et al.^3^ proposed a lung nodule segmentation algorithm using a regression neural network (RNN) for CT images. Jiantao et al.^18^ introduced a new strategy called "break and repair" shape analysis to segment lung parenchyma and nodules. An adaptive thresholding method was used to segment lung parenchyma. A size-based classification rule was applied to remove image noise from the CT images. The marching cube algorithm (MCA) was used for the geometric modelling of the lungs. Principal curvature analysis was adopted to reduce the segmentation errors. A radial basis function (RBF) was used to obtain a closed surface boundary of the lung.

Diciotti et al.^19^ reported a process for segmenting small lung nodules attached to the pleural region or blood vessels. Mukhopadhyay et al.^20^ reported a segmentation method for diagnosing lung cancer by using CT images. The VOI was selected from the lung CT images. Thresholding, connected component analysis, morphological closing, and fitting of ellipsoid techniques were performed as part of pre-processing in solid/part-solid nodule segmentation. For non-solid nodule segmentation, thresholding, connected component analysis, anisotropic diffusion filtering, and fitting of the ellipsoid technique were performed. Blood vessels attached to the nodules were removed using vascular pruning.

Abbas et al.^21^ proposed a methodology for segmenting lung nodules in CT images. pre-processing techniques such as unsharp energy masks and discrete wavelet transform have been used to enhance the image. The proposed system had an Area Overlap Measure (AOM) of 95%, Combined Equal Importance (CEI) of 92%, Hausdorff distance (HDD) of 91%, and Hammoude Distance (HMD) of 87%. Long et al.^22^) developed a fully convolutional neural network (FCN) for semantic segmentation. The fully connected layers of the CNN were removed and deconvolution layers were introduced to brand the output dimension to be the same as that of the input image. Skip connections were introduced, which combined coarse and delicate layers to make dense predictions possible. Ronneberger et al.^23^ modified and extended FCN and designed a new U-Net architecture for biomedical segmentation. The uniqueness of the UNet is that the network can segment precisely with fewer training images.

Wang et al.^24^ developed a data-driven, semi-automatic, centrally focused CNN for segmenting lung nodules. This requires improvements in segmenting nodules with sizes of approximately 3–10 mm. The DSC of this model is 82.15%, IOU is 71. 16, sensitivity is 92.75% and precision is 75.84%. Qin et al.^25^ employed a conditional generative adversarial network (CGAN) to increase the number of nodule images. Features were computed using Local Binary Pattern (LBP), Sobel, and edge operators from the synthetic nodule images, and these features were incorporated into a 3D CNN model comprising residual units. The DSC of this model is 84.83%, sensitivity is 85.11% and precision is 88.95%. Liu et al.^26^ planned a cascaded dual-pathway residual network in which the different features of many nodule types were segmented. The DSC of this model is 81.58%, sensitivity is 87.3% and precision is 79.71%.

Cao et al.^27^ developed a dual branch residual network that can simultaneously collect multi-view and multi-scale features of various nodules. The intensity features of the block's center voxel are extracted via central intensity pooling. The bounding box is manually employed to locate the nodule region, which is also a constraint in this study. The reported DSC of this method is 82.74%, sensitivity is 89.35% and precision is 79.64%.

Wu et al.^28^ developed a dual branch network based on UNet for segmenting the lung nodules. To improve the contrast between the nodules and the background, a technique called histogram equalization is applied. Global threshold binarization is used to isolate the lung parenchyma from the thoracic cavity. To locate the nodule region, the region growing approach is applied. The reported DSC of this method is 83.16%, sensitivity is 88.51% and precision is 78.98%.

Many image processing and deep learning algorithms have been proposed to solve the lung nodule segmentation problems. However, in most circumstances, algorithms that work well for one type of nodule segmentation may not be applicable to other types of nodules, regardless of how minor the differences are. Developing new architectures that operate automatically without manual intervention to segment various types of nodules is a plausible solution.

## Materials and methods

Lung_PAYNet, a deep learning architecture, has been proposed for segmenting lung nodules such as well-circumscribed, juxta-pleural, and juxta-vascular nodules. The performance of the proposed Lung_PAYNet was compared to that of a popular medical image segmentation network called UNet.

### Dataset used

The Lung Image Database Consortium and Image Database Resource Initiative (LIDC-IDRI) contains thoracic CT scans with marked-up annotated lesions for diagnosis and lung cancer screening. It is a global resource for developing new CAD systems and training and assessing existing CAD approaches for lung cancer detection and diagnosis that are openly accessible via the internet^4^.

This database, which contains 1018 cases, was created by eight medical imaging firms and seven academic centers. Each subject contained images from a clinical thoracic CT scan and an XML file that contained the results of a two-phase image annotation process performed by four thoracic radiologists. During the initial blinded-read phase, each radiologist independently assessed each CT scan and labeled lesions as "nodule > or = 3 mm," "nodule 3 mm," or "non-nodule > or = 3 mm" in one of three categories. Each radiologist independently assessed their marks and the anonymized marks of the other three radiologists in the unblinded reading phase before making the final decision.

### Region of interest extraction

In medical image analysis, extracting Region of Interest (ROI) plays a significant role. It is defined as a portion of an image regarded as significant and used for further analysis. In this research, detecting candidate lung nodules from the CT image is considered the extraction of ROI. The size of the lung CT image is 512 × 512, that consists of blood vessels, air sacs, bronchioles, sternum, aorta, superior vena cava, trachea, esophagus, spine, spinal cord, etc. If the entire lung region is used for subsequent analysis, it requires many image processing steps, and it leads to more computation resources. YOLOv3 is a popular data-driven object detection algorithm, and in this work, it has been customized and employed to detect the ROI encompassing the lung nodules from CT scans. This deep learning architecture has seven residual blocks for extracting the deep features and seven convolutional layers for generating the bounding box to locate the nodule region. Three anchor boxes with dimensions 128 × 128, 64 × 64, 32 × 32 were designed to detect different size nodules. Due to these different grids, most nodules have been detected, and they are confined within the bounding boxes. The output image of the YOLOv3 network includes the detected nodule with a bounding box and the confidence score. However, simply having a bounding box will not be enough. To accurately predict the chance of malignancy, radiologists must measure the change in nodule size in clinical settings, which necessitates manual delineation of nodule boundaries^3^.

Radiologists need manually segmented nodules at the pixel level, which takes time because nodules vary in size with diameters ranging from 3 to 30 mm, morphology, brightness, and compactness. As a result, developing a CAD system for accurate and reliable nodule segmentation is vital.

The experimental result shows that the customized YOLOv3 can detect lung nodules with high sensitivity and fewer FPs. The detected nodules have been cropped and resized to 64 × 64 patches using the inter-cubic interpolation method for further analysis. A sample nodule patch has been shown in Figure 4. These 64 × 64 nodule patches are the input for the segmentation algorithms. The advantage of generating the ROI from the 512 × 512 CT image reduces the computation burden for segmentation algorithms.

### Proposed model

The proposed Lung_PAYNet architecture is an advanced architecture based on UNet architecture. UNet, a fully convolutional neural network developed by Ronneberger et al., was introduced for segmenting medical images^23^. This network addressed two domain-specific challenges. First, traditional CNNs with fully connected layers require a large dataset, because they have a significantly high number of parameters to learn. There is a lack of large-volume datasets in the medical image domain^29–31^, which is a problem addressed by this architecture because it is very effective even when working with small datasets. Second, the UNet architecture addresses the challenge of accurately capturing context and localizing lesions at various scales and resolutions.

The proposed Lung_PAYNet represents a Pyramidal Attention Y Net for lung nodule segmentation. This architecture was proposed to make the best use of the effects of the global contextual information for semantic segmentation. The attention mechanism is paired with a spatial pyramid to extract exact dense features for pixel labelling, which is different from previously proposed segmentation architectures. Owing to the introduction of a pyramidal attention block^32,33^ on the bridge, this network can segment nodules of different sizes using high-performance metrics.

In addition, instead of convolution blocks, inverted residual blocks are adopted in the encoders and decoders to obtain features from the image. The inverted residual block is opposite to the residual block, and uses the squeeze-excitation method to give more weight to particular channels. When a CNN generates an output feature map from a convolutional or residual block, all the channels are equally significant. However, instead of treating each channel equally, squeeze and excitation techniques assign each channel-variable weighting. Furthermore, depth-wise separable convolution was used in this inverted residual block, which reduced the number of trainable parameters.

**Figure 2:**
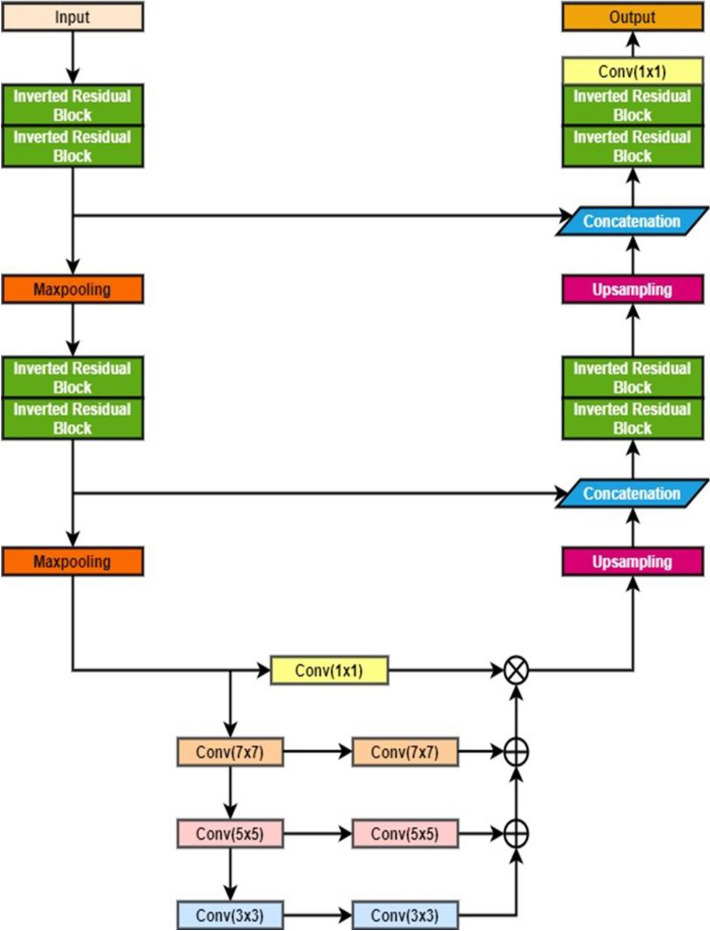
Architecture of Lung_PAYNet.

**Figure 3:**
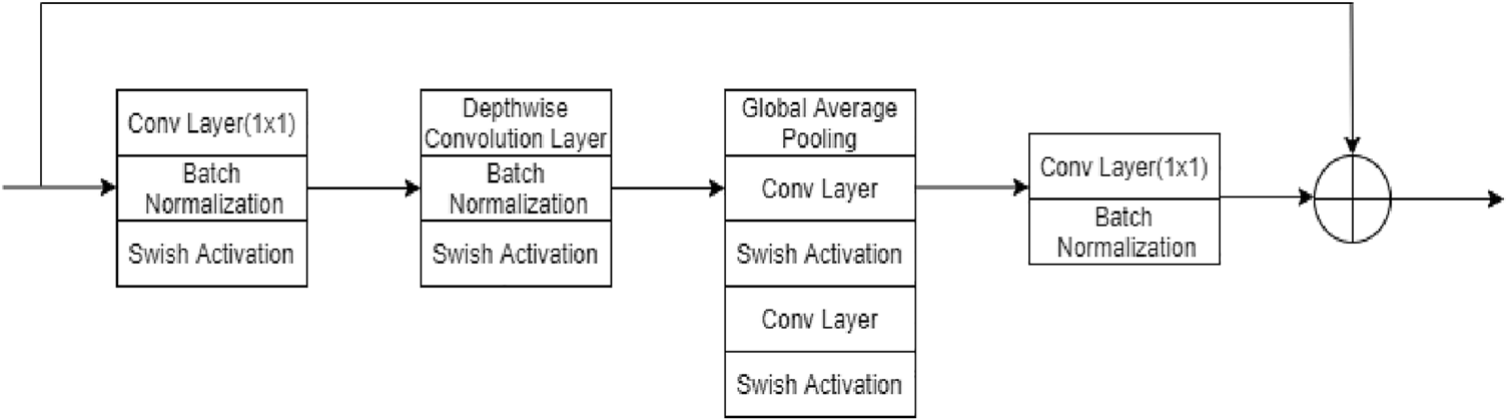
Layers of an Inverted Residual Block.

The architecture of Lung_PAYNet is shown in Fig. 2. It consists of two encoders, two max-pooling layers in the contracting path, two decoders, two upsampling layers, and a 1 × 1 convolutional layer in the expanding path. Each encoder consisted of an arrangement of two inverted residual blocks, and each decoder contained a sequence of two inverted residual blocks. The layers of an inverted residual block employed in the architecture are given in Fig. 3.

An inverted residual block was introduced in the MobileNetv2 architecture following the narrow-wide-narrow approach^34^. Narrow layers are connected via skip connections and wider layers are available inside the narrow layers. Because the ensuing 3 × 3 depth-wise convolution considerably decreases the number of parameters, the initial phase uses a 1 × 1 convolution to expand the network. Subsequently, another 1 × 1 convolution squeezes the network to match the number of channels to which it begins. Thus, the inverted residual block can extract deep features from a given input image, and the total number of parameters is low, which reduces the network training time.

The convolution operation operates on input and output channels and on the feature maps’ spatial dimension. Convolutional layers, which are commonly used in deep learning networks, have high computational costs, as shown in Eq. 1.

1 $$ {\text{H}}_{{\text{i}}} \cdot{\text{ W}}_{{{\text{i}} }} \cdot{\text{ D}}_{{\text{i}}} \cdot{\text{ D}}_{{\text{j}}} \cdot{\text{ f }}\cdot{\text{ f}} $$

where H·W represents the dimension of the input feature map; D_i_ · D_j_ represents the number of input and output channels and f · f represents the filter size.

The depth-wise convolutions performed in the inverted residual blocks mapped a single convolution on each input channel individually. Hence, the number of output channels is equal to the number of input channels. The computational cost of depth-wise convolution is shown in Eq. 2.

2 $$ {\text{H}}_{{\text{i}}} \cdot{\text{ W}}_{{{\text{i}} }} \cdot{\text{ D}}_{{\text{i}}} \left( {{\text{f}}^{{2}} + {\text{ D}}_{{\text{j}}} } \right) $$

The proposed network employed f = 3 in the inverted residual block, and the computational cost was reduced by a factor of f^2^D_j_/(f^2^ + D_j_), which was approximately nine times smaller than that of the ordinary convolution operation^35^.

The activation function employed in the inverted residual block is the swish activation function. Since the swish activation function does not have a dying neuron problem like ReLU, it is chosen to design proposed architecture.

The contracting and expanding paths are connected by a bridge consisting of a pyramidal attention network^32^. This network collected dense pixel-level attention data from the output of the contraction path.

To improve dense prediction, the pyramidal attention block uses pyramidal pooling with convolution layers with 7 × 7, 5 × 5, and 3 × 3 filters to provide meticulous attention to the spatial information of the pixels in the feature map. Pyramidal attention provides pixel-wise contextual information multiplied by the original feature map of the contracting path. It was trailed by upsampling and the fine feature map was concatenated with the coarse feature map. This process was repeated, and the decoder output was convolved with a 1 × 1 convolution filter that employed a sigmoidal activation function to produce the segmentation output.

## Experiment, results and discussions

The experiment was conducted in an NVIDIA Titan RTX GPU, and it was implemented in Keras API with a Tensorflow backend. Lung CT images were obtained from the publicly available LIDC-IDRI dataset^4^.

**Figure 4:**
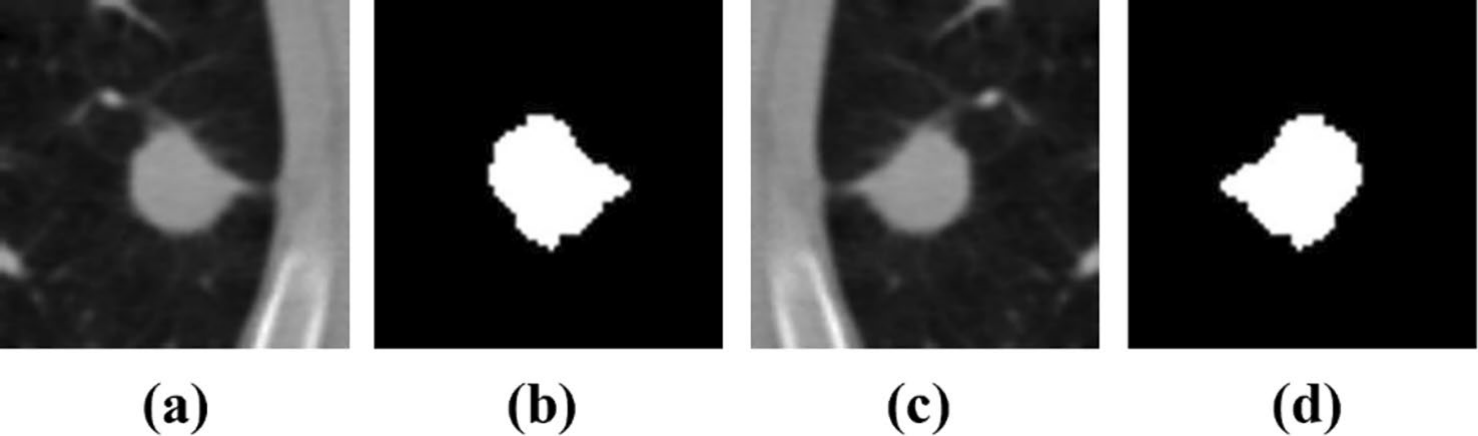
Sample Nodule Patch and Augmented Nodule Patch with Ground Truths. (**a**) Nodule Patch (**b**) Ground Truth (**c**) Augmented Nodule Patch (**d**) Augmented Ground Truth.

The dataset contains 2625 nodules, and the nodule patch had a size of 64 × 64 pixels. A data augmentation technique was employed to enhance the number of samples and reduce overfitting. The number of input nodule patches was increased by using a data augmentation method called horizontal flipping. The number of nodule patches has been increased to 5250, and out of 5250 examples, 80% of data (4200) were used for training, and 20% of data (1050) were kept for testing. Figure 4 shows a sample nodule patch, corresponding ground truth, augmented nodule patch, and the corresponding augmented ground truth^36^.

The performance of deep learning models for semantic segmentation can be measured using a variety of metrics. The (DSC) and Intersection Over Union (IOU) are the primary performance metrics for evaluating segmentation outputs. The DSC measures the connection between the ground truth and segmentation results. IOU is a straightforward metric that has proven to be successful. Jaccard index is another term. The IOU is calculated by separating the area of overlap between the predicted segmentation and ground truth by the union area between the two.

Sensitivity and precision were employed as the secondary performance metrics to verify the robustness of the evaluation. The proportion of detected pixels belonging to the nodule to all pixels of the ground-truth nodule was defined by sensitivity. Sensitivity measures the capacity of a network to segment nodules. The rate of accurately predicted pixels was measured using the precision. These metrics were calculated using Eqs. (3)–(6).

3 $$ DSC = \frac{{2*R\left( {G_{t} \cap P_{r} } \right)}}{{G_{t} + P_{r} }} $$

4 $$ IOU = \frac{{R\left( {G_{t} \cap P_{r} } \right)}}{{R\left( {G_{t} \cup P_{r} } \right)}} $$

5 $$ Sensitivity = \frac{{R\left( {G_{t} \cap P_{r} } \right)}}{{G_{t} }} $$

6 $$ Precision = \frac{{R\left( {G_{t} \cap P_{r} } \right)}}{{P_{r} }} $$

where $R$ represents Region; G_t_ represents ground truth pixels; P_r_ represents predicted segmented portion pixels.

The proposed architecture was trained with 4200 input images of size 64 × 64 pixels, and the weights were initialized using the He-normal technique. To train the proposed network, the batch size was set to 32 and the number of epochs was set to 200. The binary cross-entropy loss function is computed during the training process, and for optimizing the loss function, the Adam optimization algorithm is used with initial momentum β1 = 0.99, and β2 = 0.999. The initial learning rate was set to 0.001 and the optimizer weight decay was set to 5e-4.

An input image of size 64 × 64 pixels was passed through encoder block 1, which consisted of two inverted residual blocks. The kernel size of the inverted residual blocks was set to 3, and 32 filters were employed. The stride was set to 1 and the squeeze excitation ratio (se ratio) was set to 0.25. The feature map obtained from encoder block 1 contained 64 pixels × 64 pixels × 32 pixels. The feature map obtained after max pooling 1 was 32 × 32 × 32. Encoder block 2 follows the configuration of encoder block 1. However, the number of filters in the inverted residual blocks is double that in Encoder Block 1. The output of maxpooling layer 2 of the contracting path is only a quarter of the input image. In addition to the feature map, a pyramidal attention module was used to collect context information.

Using the 3-level pyramid, the filters can cover large, medium, and small portions of the image, and all the feature maps are fused. This pyramidal attention module output is multiplied by the feature map from the contraction path through a 1 × 1 convolution layer. The resultant feature map has dimensions of 16 × 16 × 64 pixels. The expansion path has upsampling layers and the feature maps from the respective encoder blocks are concatenated with the respective fine feature maps present in the expansion path. After up-sampling, the height and width of the image increased, whereas the depth remained constant. The resultant feature map after upsampling layer 1 was 32 × 32 × 64. After concatenating the upsampling layer 1 output with the encoder-side feature map, the resolution of the resultant feature map was 32 × 32 × 128. The number of filters of the inverted residual blocks employed in decoders 1 and 2 was 64 and 32, respectively, and the depth of the feature map was reduced by half. The feature map of decoder 1 was 64 × 64 × 32. It was passed through a 1 × 1 convolution layer with a sigmoidal activation function, resulting in a segmented output with a size equivalent to that of the input image. Table 1 lists the feature map details for Lung_PAYNet.

**Table 1:** Feature Map Details of Lung_PAYNet.

Level	Operator	Feature map
Level 1	Encoder block 1	64 × 64 × 32
	Maxpooling layer 1	32 × 32 × 32
Level 2	Encoder block 2	32 × 32 × 64
	Maxpooling layer 2	16 × 16 × 64
Level 3	Pyramidal attention block	16 × 16 × 64
Level 4	Upsampling layer 1	32 × 32 × 64
	Concatenation	32 × 32 × 128
	Decoder block 1	32 × 32 × 64
Level 5	Upsampling layer 2	64 × 64 × 32
	Concatenation	64 × 64 × 64
	Decoder block 2	64 × 64 × 32
Level 6	Convolutional layer (1 × 1)	64 × 64 × 1

The first issue in segmenting lung nodules using the UNet architecture is that the presence of nodules of various sizes attached to other structures complicates pixel classification. A pyramidal attention block is used to overcome this issue. This block can efficiently enhance the receptive field and improve the dense spatial prediction. Hence, Lung_PAYNet can segment small nodules, medium-sized nodules, large nodules, and nodules attached to the blood vessels and pleural wall. Another difficulty in UNet-based lung nodule segmentation is that convolution requires considerable computational power to learn the features of an input image. To address this problem, an inverted residual block is introduced in Lung_PAYNet, which has considerably fewer trainable parameters than UNet, thus minimizing the model's training time.

The number of trainable parameters of Lung_PAYNet was 891,713, which was much less than that of UNet, and it took 41.69 min to complete the training process of the network. The inference time for an image when testing the Lung_PAYNet model with the test image was 0.38 s. The number of trainable parameters of UNet was 8,629,921, and the training time was 451.36 min. When testing the UNet model with the test image, the inference time for the image was 1.72 s.

The training, validation, and testing settings for UNet and Lung_PAYNet were identical to ensure a meaningful comparison. The performance metrics of the proposed lung _PAYNet and UNet models are presented in Table 2.

**Table 2:** Performance Metrics of Lung_PAYNet.

Model	DSC (%)	IOU (%)	Sensitivity (%)	Precision (%)
UNet	81.94	69.4	74.12	86.28
Lung_PAYNet	95.7	91.75	92.57	96.75

**Figure 5:**
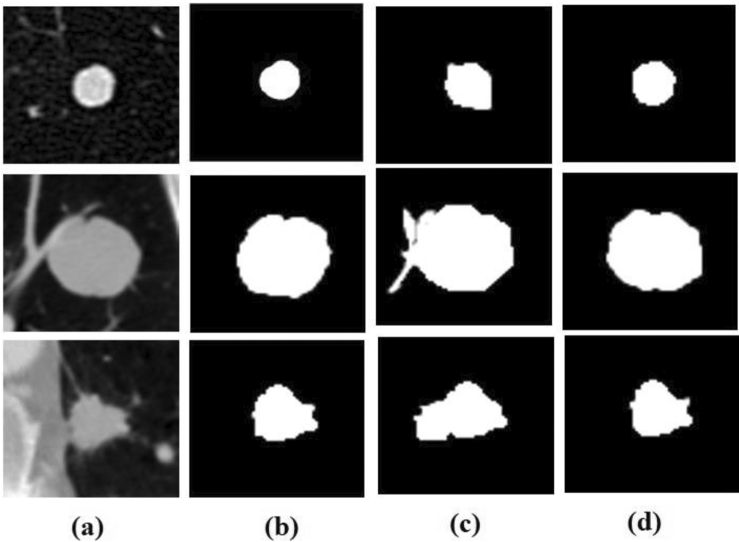
(**a**) Sample ROI Images (**b**) Ground Truths (**c**) UNet Outputs (**d**) Lung_PAYNet Outputs.

**Figure 6:**
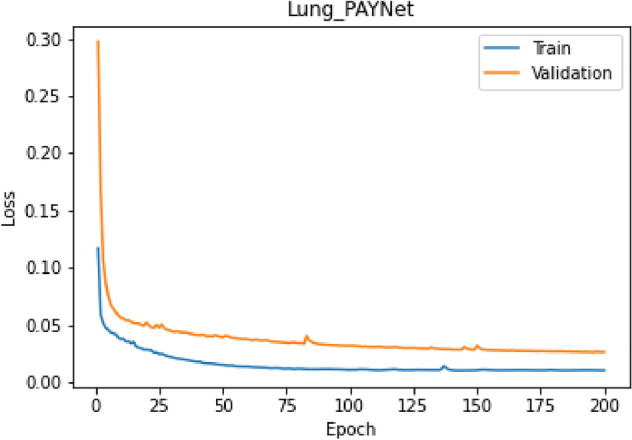
Training and Validation Losses of proposed Lung_PAYNet.

The proposed Lung_PAYNet architecture provided promising results, with 95.7% of DSC, 91.75% of IOU, 92.57% of sensitivity, and 96.75% of precision. The Lung_PAYNet showed a 13.76% improvement in DSC, 22.35% increase in IOU, 18.45% improvement in sensitivity, and 10.47% improvement in precision compared to UNet. The segmented outputs of UNet and Lung_PAYNet are shown in Fig. 5. Most of the nodule (foreground) pixels were correctly segmented for different nodules, such as juxta-vascular, well-circumscribed, and juxta-pleural nodules, in the case of the Lung_PAYNet model, and this network can segment nodules of different sizes. Figure 6 shows the training and validation losses of the proposed lung _PAYNet model. It is observed that the model is neither overfitting not underfitting.

Table 3 compares the performance metrics of the planned architectures with those of state-of-the-art lung nodule segmentation techniques. It should be noted that the proposed architecture showed promising results for lung nodule segmentation.

**Table 3:** Comparison of the Proposed Architectures with State-of-the-Art Segmentation Techniques.

Model	DSC (%)	IOU (%)	Sensitivity (%)	Precision (%)
Proposed Lung_PAYNet	95.7	91.75	92.57	96.75
UNET	81.94	69.4	74.12	86.28
Central focused CNN (Wang et al.)^24^	82.15	71.16	92.75	75.84
3D UNET with LBP, Sobel, Canny operators (Qin et al.)^25^	84.83	–	85.11	88.95
Cascaded dual pathway residual network (Liu et al.)^26^	81.58	–	87.3	79.71
Dual branch residual Network with central intensity pooling (Cao et al.)^27^	82.74	–	89.35	79.64
Dual branch UNET with region growing algorithm (Wu et al.)^28^	83.16	–	88.51	78.98

Wang et al.^24^ reported a centrally focused CNN model for segmenting lung nodules that used 2D and 3D lung nodule patches for training. A central pooling layer was implemented to save many patch center features, while eliminating unnecessary patch edge features. A weighted sampling strategy was devised to identify problematic nodule voxels. To lower the computing cost of the network, nodule patches of size 50 × 50 were constructed from 512 × 512 lung CT slices. A nodule patch is manually generated, which is a drawback of this method. This model is capable of segmenting well-defined nodules and juxta-pleural nodules but not juxta-vascular nodules. The DSC of this model was 82.15%, which is 13.55% lower than Lung_PAYNet.

Qin et al.^25^ proposed a segmentation method that uses local binary pattern maps to represent nodule texture, and Sobel and Canny detectors to represent edge maps. These maps can be considered as preliminary segmentation results that were refined using the 3D CNN architecture. When texture and edge features are manually incorporated into a CNN, the model loses its generalization potential and restricts its use in clinical practice. This technique had a DSC of 84.83%, which is lower than Lung_PAYNet by 10.87%.

Liu et al.^26^ suggested a segmentation method called dual-path structure based on the residual mechanism for extracting context information. However, this method is a semi-automated segmentation method because the nodule location must be specified manually. The reported DSC of this method was 81.58%, which is 14.12% lower than Lung_PAYNet.

Cao et al.^27^ developed a dual-branch residual network that could simultaneously collect multi-view and multiscale features of various nodules. The intensity features of the center voxel of the block were extracted using central intensity pooling. A weighted sampling technique based on nodule boundaries was used to select the border voxels. Juxta-vascular nodules could not be segmented using this method. The weighted sampling approach, on the other hand, produces poor sampling results for small nodules. A bounding box was manually employed to locate the nodule region, which is also a constraint in this study. The reported DSC of this method was 82.74%, which is 12.96% lower than Lung_PAYNet.

Wu et al.^28^ developed a dual-branch network created on UNet to segment lung nodules. To improve the contrast between the background and nodules, a technique called histogram equalization was applied. Global threshold binarization was used to isolate lung parenchyma from the thoracic cavity. A region-growing approach was used to locate the nodules. A dual-branch UNet was utilized for fine segmentation after roughly finding lung nodules. The model lacks generalization capability because it requires many image-processing steps before extracting deep features from nodules. The threshold and initial seed values were defined by the user based on the input image. These are limitations of the segmentation network proposed by Wu et al.^28^. The reported DSC of this method was 83.16%, which is 12.54% lower than Lung_PAYNet.

## Conclusion

The proposed Lung_PAYNet architecture shows a robust capability to autonomously study lung nodule-sensitive features from CT images. The model demonstrated encouraging segmentation results for the nodules with diverse characteristics. Lung_PAYNet was proposed with fewer layers to obtain a better segmentation output for all types of nodules with fewer computational resources. Owing to the depth-wise convolution operation in the inverted residual blocks, Lung_PAYNet can learn lung nodule features with fewer parameters, thereby reducing the computational burden of the network and eliminating overfitting. Moreover, the pyramidal attention block with different kernel sizes gives the network precise attention to the nodule's spatial details, and the segmentation efficiency is appreciable for challenging nodules such as juxta-vascular and juxta-pleural nodules.

## Data Availability

The datasets used in this paper are open access data available from the link indicated in ref^4^ Cancer Imaging Archive. Accessed: Jan.5, 2020. [Online]. Available: https://wiki.cancerimagingarchive.net/display/Public/LIDC-IDRI.
